# Research advances in Ecuador on use of entomopathogenic fungi for control of the cattle tick, *Rhipicephalus microplus*: the case of *Beauveria bassiana* sensu lato strain INIAP L3B3

**DOI:** 10.3389/ffunb.2025.1492395

**Published:** 2025-04-03

**Authors:** David Hidalgo, José Luis Ramírez, Mercedes Navarrete, Víctor Cevallos, Mario Ramos, Bill Bravo, Klever Carranza, Víctor Montes, Adalberto Á. Pérez de León

**Affiliations:** ^1^ Laboratory of Plant Protection, Instituto Nacional de Investigaciones Agropecuarias (INIAP), Santo Domingo Research Station, La Concordia, Ecuador; ^2^ Crop Bioprotection Research Unit, National Center for Agricultural Utilization Research, United States Department of Agriculture-Agricultural Research Service (USDA-ARS), Peoria, IL, United States; ^3^ Facultad de Veterinaria, Universidad Técnica de Manabí, Portoviejo, Ecuador; ^4^ San Joaquin Valley Agricultural Sciences Center, USDA-ARS, Parlier, CA, United States

**Keywords:** *Beauveria bassiana*, mycopesticide, cattle tick, *Rhipicephalus microplus*, microbial control

## Abstract

Ecuador is one of the countries in the world where ticks and tick-borne diseases are major constraints on cattle health and productivity. The intense use of synthetic acaricides to manage tick infestations resulted in widespread acaricide resistance in the tick *Rhipicephalus microplus*, which is known to infest over 75% of the farms where cattle are raised in the country. Sustainable and environmentally friendly alternatives to control the cattle tick *R. microplus* are needed urgently. This minireview describes a project at the Santo Domingo Experimental Station of the National Institute of Agricultural Research to develop biopesticides for tick management to exemplify advances in collaborative research on the use of entomopathogenic fungi as active ingredients, or mycopesticides, in formulations to control *R. microplus*. Research and development conducted in multiple phases revealed the *in vitro* and *in vivo* acaricidal properties of *B. bassiana* sensu lato (s.l.) strain INIAP L3B3. These efforts followed efficacy and safety norms issued by the government agency of Ecuador in charge of controls and regulations to protect an improve animal health, plant health, and food safety. Results described herein indicate that *B. bassiana* s.l. INIAP L3B3 can be registered as an eco-friendly mycopesticide alternative to synthetic chemical acaricides or could complement conventional chemical acaricide applications for integrated *R. microplus* management programs in support of sustainable cattle raising in Ecuador.

## 1 Introduction

Different species of entomopathogenic fungi have lethal effects on the tick *Rhipicephalus microplus* (López et al., 2009; Cafarchia et al., 2022), which is considered the most economically important hematophagous ectoparasite and vector of pathogens causing significant morbidity and mortality among cattle herds globally (Pérez de León et al., 2020). Like in neighboring countries of South America where this cattle tick is present (Rodríguez-Hidalgo et al., 2017), livestock farmers in Ecuador also face widespread resistance to synthetic acaricides, which are extensively used to control ticks including *R. microplus* (Lopez-Arias et al., 2014; Rodríguez-Hidalgo et al., 2017; Pérez-Otáñez et al., 2024c; Pérez-Otáñez et al., 2024a). Widespread resistance to synthetic acaricides highlights the need for alternative solutions, such as the development and commercialization of biopesticidal formulations utilizing entomopathogenic fungi as active ingredients, or mycopesticides (Jiang and Wang, 2023). Eco-friendly technologies like mycopesticides present a promising method for integrated *R. microplus* management (Angelo et al., 2022). However, there is a need to innovate mycopesticide formulations that are highly virulent, cost-effective, and stable (da Paixão et al., 2023; Friuli et al., 2023; da Silva Medina et al., 2024). Such advancements could enhance the commercialization and use of mycopesticides with other technologies to manage the parasitic and non-parasitic life stages of *R. microplus* infesting cattle and pastures, respectively. Relative to other countries in South America (Fernandes and Bittencourt, 2008; Moncada et al., 2015), research and development of effective and safe entomopathogenic fungi formulations commercially available for use under field conditions to control *R. microplus* in Ecuador remains to be fully realized.

**Figure 1:**
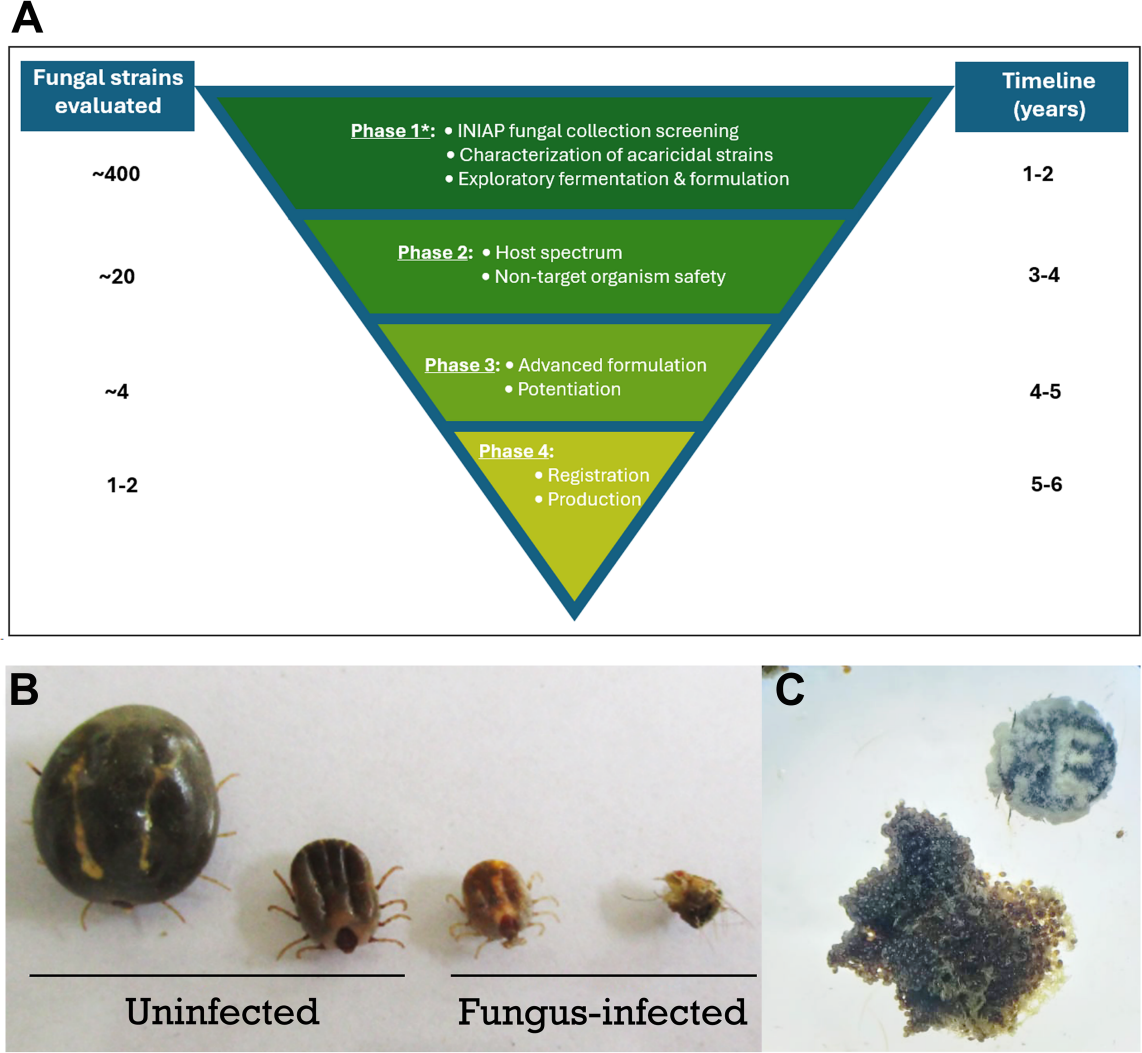
**(A)** Theoretical research and development approach to register and commercialize a mycopesticide formulation to manage the cattle tick *Rhipicephalus microplus* in Ecuador. *Adapted from Hidalgo (Hidalgo Mata et al., 2019). Phase 1: concept based on pre-existing collection including entomopathogenic fungi; Phase 2: includes mammalian and non-target organism safety determinations; Phase 3: includes photo and thermostability optimization, determination of synergy with other pesticides; Phase 4: adheres to federal regulatory framework of Ecuador to register and commercialize agricultural products (https://www.agrocalidad.gob.ec/?page_id=39111). **(B)** Phenotypic characteristics in *R. microplus* ticks following infection with *B*. *bassiana* s.l. INIAP L3B3, note color and size changes in fungus-infected ticks. **(C)** Adult tick cadaver in upper right corner, and tick egg-mass in lower left section, covered with mycelial growth.

This minireview highlights collaborative efforts by Ecuador’s National Institute of Agricultural Research (INIAP), particularly at the Santo Domingo Experimental Station (INIAP-SDES), through the project “Isolation, Maintenance, Reactivation, and Pelletizing of Entomopathogenic Fungi”. An objective of this project is to develop biopesticides based on entomopathogenic fungi to control ticks (INIAP, 2021). Previous studies in Ecuador documented the utility of entomopathogenic fungi to control *R. microplus* testing experimental mycopesticide formulations containing *Lecanicillium lecanii*, *Metarhizium anisopliae*, or *Beauveria bassiana* (Navarrete et al; Piguave, 2016; Morocho, 2019; Lopez and Masiel, 2021). However, cattle producers in Ecuador still lack a registered mycopesticide product that can be used with other technologies for integrated *R. microplus* management in their farms. **Figure 1A** provides an overview of the research and development pathway for registering and commercializing a mycopesticide product facilitated by the collection of Ecuadorian entomopathogenic fungi at INIAP (Arahana et al., 2013). This collection enabled bioassays to identify strains with acaricidal properties. Research progress with the INIAP-SDES project to deliver a registered mycopesticide product for *R. microplus* control is described below.

## 2 Tick model selection

*R. microplus* is the most common tick infesting cattle in Ecuador (Pérez-Otáñez et al., 2024b). It is estimated that over 75% of the cattle farms in the country are infested with *R. microplus* (Vizcarra et al., 2015). In addition to direct blood feeding effects associated with its parasitic lifestyle, *R. microplus* also transmits pathogens like *Babesia bovis*, *B. bigemina*, and *Anaplasma marginale* that impact the health and diminish the productivity of cattle herds in Ecuador (Maya-Delgado et al., 2020). Acaricide resistance complicates *R. microplus* control based on chemical treatments with synthetic acaricides widely available to farmers (Paucar et al., 2022). Thus, the INIAP-SDES project targeted *R. microplus* to develop biopesticides based on entomopathogenic fungi for tick control.

## 3 Phase 1: selection of entomopathogenic fungal strains active against *R. microplus*

From the collection mentioned above, 80 fungal isolates obtained from a field survey with palm weevil (*Rhynchophorus palmarum*) traps were of particular interest. Bioassay of these isolates with the rice weevil (*Sitophilus oryzae*) yielded nine entomopathogenic strains native to the province of Santo Domingo de los Tsáchilas where the majority of the territory has a tropical climate with parts to the east in the Andean foothills with temperate climate (INIAP, 2021). *B. bassiana* s.l. strains INIAP L3B3 and INIAP O2 were selected for their activity against *R. microplus* (**Figures 1B, C**).The INIAP O2 strain was dropped from further testing because of lower activity against *R. microplus* than the L3B3 (data not shown). Fungal strains were propagated using rice as the solid substrate. However, during the initial bioassays with *B. bassiana* s.l. INIAP L3B3 it was observed that, despite a notable increase in conidia production, the highest mortality achieved was 8.3% on day 9 post-treatment at a concentration of 6.9 x 10^6^ conidia/ml. Suspecting loss of virulence, the experiments were repeated using fungi recovered from tick larva cadavers that had sporulated. Following this reactivation, the median lethal dose (LD50) of *B. bassiana* s.l. INIAP L3B3 was 1.29 x 10^4^ spores/ml (Herrera Montero et al., 2024). The molecular characteristics of *B. bassiana* s.l strain INIAP L3B3 was provisionally identified through Sanger sequencing of the ITS gene (unpublished data). Comprehensive characterization is yet to be completed.

**Figure 2:**
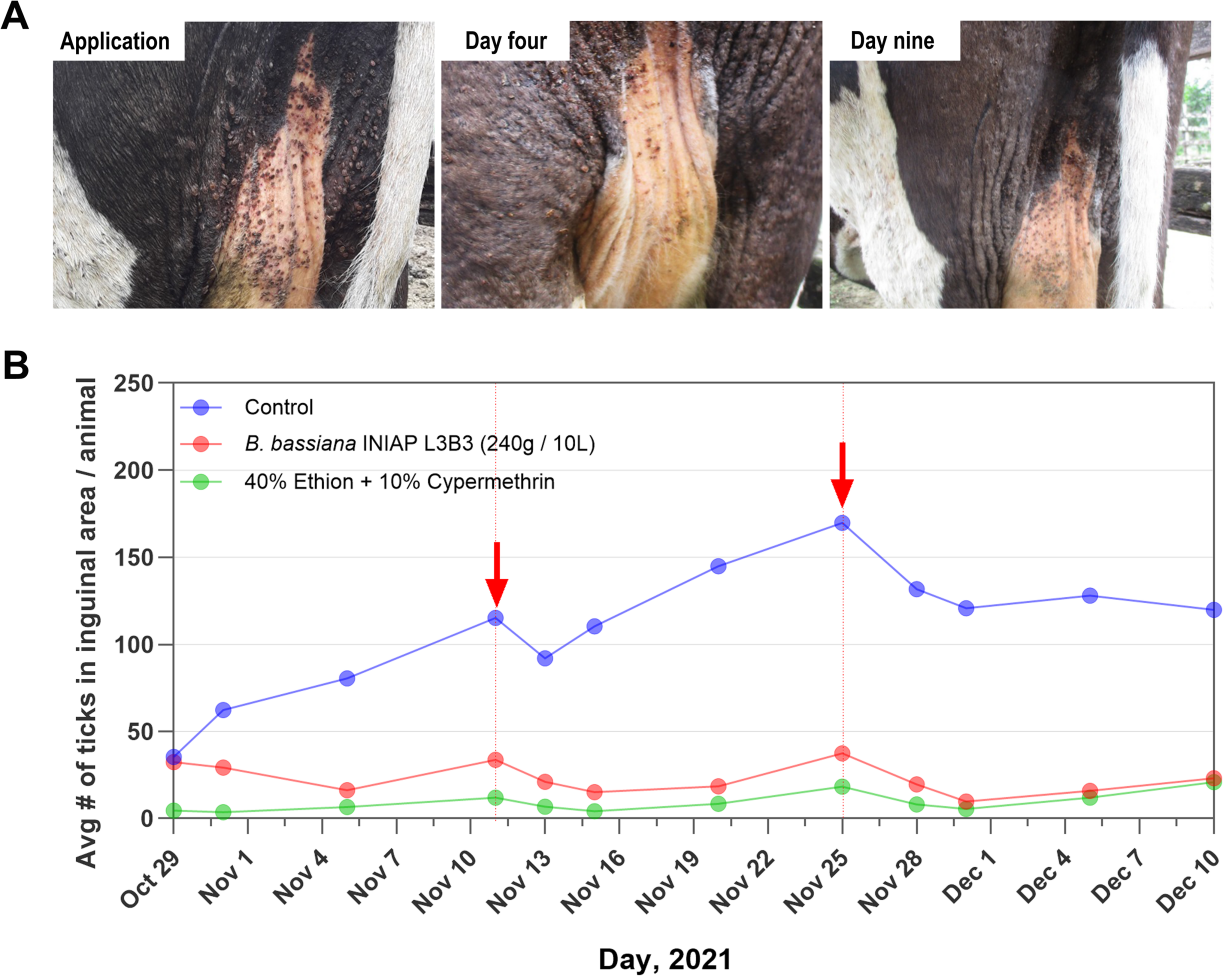
**(A)** Drying and detachment of ticks following treatment with entomopathogenic fungal strain *B*. *bassiana* s.l. INIAP L3B3. Photos of the same bovine through time depicting reduction of tick infestations following treatment with *B*. *bassiana* s.l. INIAP L3B3 experimental formulation. **(B)** Temporal effect on average number of *R. microplus* ticks in each group of cattle sprayed with *B*. *bassiana* s.l. INIAP L3B3, commercial chemical acaricide mixture of 40% Ethion + 10% Cypermethrin, or water (Control). Dots represent the average number of ticks counted in inguinal area for each of the 3 cattle groups (n= 5) on the date shown. Red arrows indicate dates of chemical acaricide or fungal re-application. Graph generated with data from Bravo and Carranza, 2022.

A farm in the area of Santo Domingo de los Tsáchilas province where populations of *R. microplus* resistant to acaricides are known to infest cattle was selected for pilot study with *B. bassiana* s.l. INIAP L3B3 (Bravo et al., 2022). These experiments are described in videos available on the INIAP online channel (INIAP, 2022; INIAP, 2023). By comparison to the effects of chemical treatment by spray with a commercially available combination of 40% ethion + 10% cypermethrin using a backpack pump, *R. microplus* started to show the effects of entomopathogenic infection as early as 4 days after cattle infested naturally were sprayed similarly with *B. bassiana* s.l. INIAP L3B3 at a dose of 240 grams/10 liters of dechlorinated water (**Figures 2A, B**). Using the average number of *R. microplus* counted in the inguinal area as indicator of the infestation level in cattle in the control group, the relative effect of repeated treatment between *B. bassiana* s.l. INIAP L3B3 and 40% ethion + 10% cypermethrin was comparable, especially after the second reapplication (**Figure 2B**). These results were obtained without using the target lethal dose of 10^7^ because by dissolving the 1.43×10^8^ conidia/g in 10 liters of water the maximal concentration of conidia sprayed on cattle was 10^6^.

Greenhouse studies revealed that *B. bassiana* s.l. INIAP L3B3 was also active against *R. microplus* eggs, thus expanding the potential use of this fungal strain (Ligña Sangucho et al., 2024). The study utilized various concentrations of conidia (ranging from 7.42x10^7^ to 7.42x10^3^ conidia/ml) applied to tick eggs in both sterile and non-sterile soil. The results showed an ovicidal efficacy of 20% at a concentration of 7.42x10^7^ conidia/ml within six to ten days, with higher efficacy in non-sterile soil. The conidia remained viable for up to twenty days, effectively controlling not only eggs but also larvae and nymphs.

## 4 Phase 2: safety assessment of *B. bassiana* s.l. INIAP L3B3

*B. bassiana* is generally regarded as safe for mammals, non-target organisms and the environment (INIAP, 2023). Scientific literature on safety evaluations of *B. bassiana* strains intended for use as active ingredients in mycopesticide products was reviewed to observe best practices handling strain INIAP L3B3 in the laboratory and the pilot field study (Cazorla Perfetti et al., 2015; Authority et al., 2020; Sayed Ali et al., 2022; Ligña Sangucho et al., 2024). Previous reports indicated that other *B. bassiana* strains were safe to treat cattle infested with *R. microplus* (Navarrete et al., 2017; Lopez and Masiel, 2021).

No adverse reactions to the water formulation containing *B. bassiana* s.l. INIAP L3B3 were observed in the sprayed cattle. Cattle appeared normal after reapplication of the *B. bassiana* s.l. INIAP L3B3 spray formulation. No health concerns associated with spraying *B. bassiana* s.l. INIAP L3B3 were reported by the farm workers that treated the cattle.

## 5 Phase 3: potentiation and advanced mycopesticide formulation

Based on previous studies documenting that acaropathogenic activity of entomopathogenic fungi could be potentiated by the synergistic effect of other substances (Marrone, 2019; da Silva Medina et al., 2024), the combined effect of *B. bassiana* s.l. INIAP L3B3 with other organic and synthetic acaricides was tested. Bioassays evaluated the control of *R. microplus* combining *B. bassiana* s.l. INIAP L3B3 with plant extracts (eucalyptus, thyme, onion, and garlic) and the synthetic acaricide mixture containing cypermethrin + ethion (Herrera Montero et al., 2024). Compatibility test between *B. bassiana* and the botanical or chemical compounds were evaluated *in vitro* prior to testing with tick larvae. These bioassays indicated that while fresh garlic extract was acaricidal, it also inhibited conidial germination when combined with *B. bassiana*. Among the botanical extracts tested, only fresh garlic extract was effective at a concentration of 45 ml/l. Combining *B. bassiana* with garlic yielded an LD50 of 1.63 x 10^4^ spores/ml, though garlic initially inhibited fungal germination until allicin, the active component, dissipated. The combination of *B. bassiana* s.l. INIAP L3B3 conidia with the chemical acaricide (cypermethrin + ethion) indicated synergy between the two (Herrera Montero et al., 2024). This resulted in exponential reduction of the LD50 for *B. bassiana* conidia from 1.14x 10^4^ when used alone to a LD50 of 1.18 x 10^3^ conidia/ml and 0.5ml/L of chemical acaricide when used in combination.

The optimization of solid-state mass production of conidia from *B. bassiana* s.l. INIAP L3B3 was explored to develop large-scale applications. Enhancement of eighteen formulations was evaluated with nutritional supplements (palm kernel meal, quinoa flour, brewer’s yeast, and powdered milk) combined with rice and barley (Romero Suarez et al., 2024). These experiments revealed significant differences in conidia production. The best substrate was 2.5% brewer’s yeast + 97.5% rice + Beauveria spp. yielding 6.9 x 10^9^ conidia/g. The highest colony-forming units (CFU) concentration achieved was 2.64 x 10^10^ CFU/g, while purity percentages did not significantly differ among treatments. These findings were applied to scale up production of *B. bassiana* s.l. INIAP L3B3 conidia. Formulation improvements ensured a high-quality mycopesticide effective against *R. microplus*.

## 6 Phase 4: registration and production

Registration of mycopesticides in Ecuador is a process that requires adherence to efficacy and safety norms issued by Agrocalidad, which is the government Agency of Ecuador in charge of controls and regulations to protect an improve animal health, plant health, and food safety. The process involves an application that includes detailed information about the applicant, the producing company, the biological characterization of the fungus, and the results of biological efficacy trials conducted in the field (Agrocalidad, 2022). Efficacy data must be generated according to the “Instructions for the Approval, Execution, and Supervision of Efficacy Tests of Pesticides and Related Products for Agricultural Use in Ecuador” issued by (Agrocalidad, 2021). Per the Instructions, trials must be supervised by authorized agencies or conducted in collaboration with universities or research centers.

The INIAP-SDES project is expanding collaboration with the National Livestock and Pasture Program of Ecuador to develop and execute confirmatory tests to treat more cattle in multiple farms with a candidate formulation of *B. bassiana* s.l. INIAP L3B3. It is expected that these confirmatory tests will generate the efficacy and safety information needed to register a mycopesticide product in Ecuador with *B. bassiana* s.l. INIAP L3B3 as the active ingredient. A plan for the commercial production of mycopesticides by SDES-INIAP was presented to the national government, and the general direction of INIAP is exploring the possibility of licensing production and commercialization to private companies.

## 7 Discussion

The INIAP-SDES project to research and develop mycopesticides for tick control described herein addresses the problem with widespread multi-acaricide resistant populations of *R. microplus* in Ecuador. *R. microplus* and associated diseases are considered the main health burden and cause of economic loss in cattle herds nationally (Vizcarra et al., 2015; Rodríguez-Hidalgo et al., 2017; Maya-Delgado et al., 2020; Pérez-Otáñez et al., 2024c; Pérez-Otáñez et al., 2024a). Dividing the project into 4 phases depicted in **Figure 1B** facilitated the organization of research and development efforts, monitoring progress, and adaptation of the strategy for completion within the projected timeline. Although progress up to Phase 3 was documented, partnership with the National Livestock and Pasture Program of Ecuador is allowing the project to be completed as planned (Bustillo Pardey and Marin, 2002; INIAP, 2022; Pérez-Otáñez et al., 2024a). The loss of virulence of the *B. bassiana* s.l L3B3 strain jeopardized the project, requiring the experiment to be repeated after reactivating the fungus on tick larvae to recover its virulence (Herrera Montero et al., 2024). The loss of virulence in entomopathogenic fungi can be caused by factors such as successive culturing without proper reactivation, inadequate storage conditions, or genetic changes. To restore the virulence of entomopathogenic fungi, it is crucial to conduct passages on the target insect and maintain strict quality control during the reactivation process, which is essential to ensure the fungus’s effectiveness in biological control applications (Bustillo Pardey and Marin, 2002; Tomilova et al., 2023). Although acaricide resistance was not confirmed in the *R. microplus* infesting cattle sprayed with the experimental *B. bassiana* s.l. INIAP L3B3 formulation, the management of resistant cattle ticks was recommended because 83% of farms sampled in Santo Domingo de los Tsáchilas province where acaricides are used intensely were infested with *R. microplus* (Pérez-Otáñez et al., 2024b). Additionally, the sustained number of ticks on the control cattle sprayed with water in the pilot study suggested that pastures in the farm were infested with the non-parasitic life stages of *R. microplus*. Field tests are needed to confirm the ovicidal activity observed in the greenhouse studies, which suggests that *B. bassiana* s.l. INIAP L3B3 formulations could be used in integrated tick management to target multiple life stages of *R. microplus*. The ovicidal characteristic is particularly relevant, as the commercial formulation of *M. brunneum* F52 used in the control of *Anoplophora glabripennis* showed an ovicidal effect of up to 40% under specific high-humidity conditions in the field (Goble et al., 2015).

Field tests are needed to confirm the synergistic effect observed *in vitro* when *B. bassiana* s.l. INIAP L3B3 was combined with the cypermethrin + ethion acaricidal mixture. This highlights the nuances of formulating acaricide mixtures with entomopathogenic fungi on acaricidal efficacy because the compatibility of *B. bassiana* can vary depending on the class of acaricide or members of the same acaricide class used in the mix (Cafarchia et al., 2022; Friuli et al., 2023). Our observations confirmed the reported acaricidal property of garlic and despite inhibiting fungal germination, *B. bassiana* s.l. INIAP L3B3 could be alternated with garlic extract to treat infested pastures (Herrera Montero et al., 2024). Advances in formulation science and technology could allow further improvements for efficient mass production of *B. bassiana* s.l. INIAP L3B3. The project is considering a pellet formulation using local inputs to make production of a high-quality *B. bassiana* s.l. INIAP L3B3-based mycopesticide more cost-effective (Hidalgo and Adrian, 2019; Hidalgo Mata and Tello Torres, 2022).

Development of a robust regulatory framework to register and control mycopesticides and other biopesticides in Ecuador since the second decade of this century provided a critical path for this project (Agrocalidad, 2021; Agrocalidad, 2022). Clarity on the registration process expedites implementation research and the translation of efficacy and safety results from laboratory and field studies (Jiang and Wang, 2023), which could accelerate the commercialization of mycopesticides for sustainable *R. microplus* control. The results obtained with this project thus far indicate that *B. bassiana* s.l. INIAP L3B3 can be registered as an eco-friendly mycopesticide alternative to synthetic chemical acaricides or could complement conventional chemical acaricide applications for integrated *R. microplus* management programs in support of sustainable cattle raising in Ecuador.
